# Metaplastic Conditions in The Bladder in Patient With Epidermolysis Bullosa

**DOI:** 10.1590/S1677-5538.IBJU.2015.0347

**Published:** 2016

**Authors:** Kenan Yilmaz, Deniz Demirci, Numan Baydilli, Sinan Nazlim

**Affiliations:** 1 Department of Pediatric Nephrology, Faculty of Medicine, Erciyes University, Kayseri, Turkey; 2 Department of Urology, Faculty of Medicine, Erciyes University, Kayseri, Turkey; 3 Department of Pathology, Faculty of Medicine, Erciyes University, Kayseri, Turkey

**Keywords:** Urinary Bladder, Epidermolysis Bullosa, Metaplasia

## Abstract

Epidermolysis bullosa is a rare inherited muco-cutaneous disorder that sometimes presents with genitourinary involvement. Herein we report the case of an 11-year-old girl with a history of junctional epidermolysis bullosa who was admitted with urological symptoms. On cystoscopy, suspected bullous bladder lesions were observed. Mesonephroid, intestinal and squamous metaplasia is reported here for the first time.

## INTRODUCTION

Epidermolysis bullosa (EB) is a rare heterogeneous dermatological disorder. In spite of the increasing rates of recent publications of patients presenting with urological complications EB has rarely been reported in the literature (1).

We were unable to find any reports of accompanying mesonephroid, intestinal or squamous metaplasia of the bladder in junctional EB in the English literature. Here we report the first case of an 11-year-old girl with the diagnosis of junctional EB with accompanying mesonephroid, intestinal and squamous metaplasia in the bladder.

## CASE REPORT

**Figure 1:**
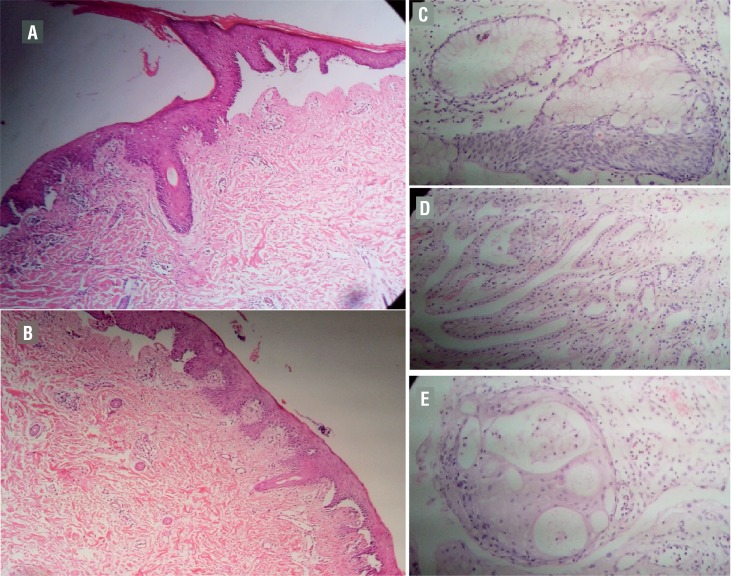
Epidermolysis bullosa patient's skin biopsy (A, B) and Epidermolysis bullosa patient's bladder biopsy. The bladder epithelium exhibits marked metaplastic changes (C, D, and E). (A, B) There are marked subepidermal blisters of noninflammatory type. The epidermis shows focal spongiosis. Mild dermal fibrosis and sparse inflammatory infiltrate in the dermis is observed. Hematoxylin and eosin stain, original magnification x100; (C) Intestinal metaplasia of the urinary bladder. A complex glandular structure lined by mucin-producing columnar cells. Hematoxylin and eosin stain, original magnification x 400; (D) Mesonephroid metaplasia of the urinary bladder. A complex clustering of microcystic and tubular formations lined by cuboidal to flattened cells in edematous stroma. Hematoxylin and eosin stain, original magnification x 400; (E) Squamous metaplasia of the urinary bladder. Note characteristics of squamous nests. Hematoxylin and eosin stain, original magnification x 400.

**Figure 2:**
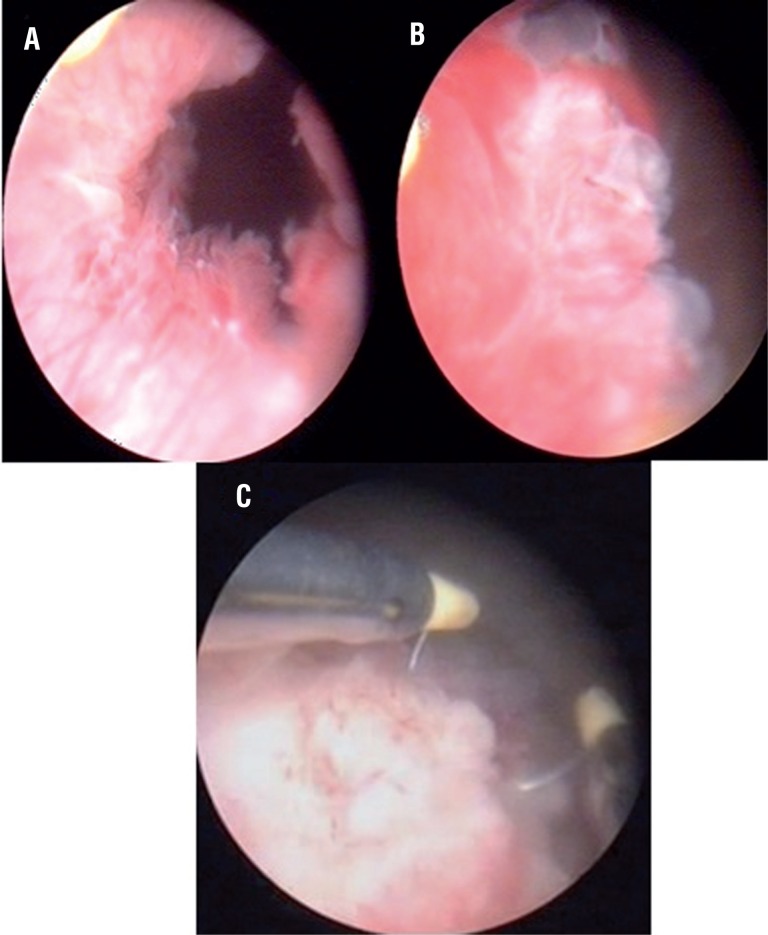
Cystoscopic images of lesions at bladder neck and right bladder wall.

An 11-year-old female patient, who was first diagnosed with junctional EB at 18 months of age, was admitted to our institution with hematuria and irritative voiding symptoms. She was under the care of dermatology clinic. Her physical examination revealed that her skin lesions were in remission. Her skin biyopsy image is shown in Figures 1A and B. However, in her urological examination, distal urethral stenosis was detected. There was microscopic hematuria. Urinary culture was sterile. Blood cell count and creatinine levels were within the normal ranges. In uroflowmetric examination there was normal outflow rate and no post voiding residue. Bladder wall thickness was shown to be increased (7mm) on ultrasound examination. Van Buren dilatation was performed for female distal urethra. Cystoscopic evaluation showed several suspicious lesions (such as papilla-bullosa) around the bladder neck and right bladder wall. A complete transurethral resection was performed, as shown in Figure-2.

Histopathological examination reported mesonephroid, intestinal and squamous metaplasia in the bladder, as shown in Figures 1 C, D, and E. After the operation, the patient's complaints were resolved. Patient was followed-up for 2 years because of the bladder lesions by cystoscopy. After 2 years, cystoscopy follow-up ended and patient is still under follow-up using ultrasound and urine analysis. She didn't have any urinary complains up to present. No recurrent lesions were detected as metaplasia.

## DISCUSSION

Epidermolysis Bullosa is a rare and severe disorder characterized by blistering lesions of the skin after trauma. There are four major type of inherited epidermolysis bullosa: EB simplex (EBS), junctional EB (JEB), dystrophic EB (DEB) and Kindler syndrome. The level of separation occurs within the epidermis in Epidermolysis bullosa simplex (EBS), in the lamina lucida in junctional EB (JEB) and under the lamina densa in dystrophic EB (DEB). The cleavage plane in Kindler Syndrome (KS) can occur within the basal keratinocytes, at the level of the lamina lucida or below the lamina densa (2). Kindler syndrome (KS) is an autosomal recessive skin disorder characterized by traumatic acral blister formation in infancy and early childhood, progressive poikiloderma, cutaneous atrophy and increased photosensitivity. This rare genodermatosis represents combination of clinic features of hereditary epidermolysis bullosa and poikiloderma congenitale (3). The first case report of genitourinary tract involvement in EB was published in 1973 by Kretkowski (4).

Our patient, who had JEB (generalized intermediate) was diagnosed based on the clinical and histopathological (with immunofluorescence) findings. After the first year of life, her non-inflammatory blistering skin lesions in areas of the body exposed to mechanical trauma went into remission for several years. However, many years later she was admitted to our clinic with dysuria and hematuria. After examination, distal urethral stenosis and a thickened bladder wall were detected by ultrasound. The clinical spectrum in the literature varies from meatal stenosis leading to upper urinary tract dilatation to serious stenosis due to bullous lesions and scarring at the ureterovesical junction requiring permanent urinary diversion (1). In our case Van Buren dilatation was preferred for meatal stenosis due to female genitalia.

Mesonephroid metaplasia is an unusual lesion confined to the lamina propria of the lower urinary tract. It is defined by a characteristic histologic picture of tubular structures, formed by a single layer of cuboidal cells, surrounded by a thick basement membrane. In large published series of cases of mesonephroid metaplasia, the lesions were in most cases associated with chronic infection, stone disease or repeat surgical intervention in the genitourinary tract. Characteristically, the epithelial cells of nephrogenic adenomas show clear cell cytoplasm with vacuoles and uniform nuclei without mitoses in the benign form. The malignant form is characterized by mitotic figures and/or invasions into the muscle (5).

Intestinal metaplasia usually occurs with long standing inflammation/irritation, such as from indwelling catheters, calculi, neurogenic bladder and bladder exstrophy and it may be focal or diffuse, but it is usually only seen microscopically. Intestinal metaplasia often co-exists with adenocarcinoma of the bladder, and some authors have proposed that intestinal metaplasia may be a precursor lesion (6).

Squamous metaplasia is clinically significant and may be associated with the development of bladder cancer, bladder contracture or obstructive uropathy. According to different studies, the risk of developing squamous cell carcinoma in patients with keratinizing squamous metaplasia is estimated to be 21 to 42% (7).

In our case there was a predisposing factor, namely chronic blistering. In our opinion mesonephroid, intestinal and squamous metaplasia were the result of inflammation in the lamina lucida in junctional EB. In addition to JEB, urological complications have also been reported such as recessive DEB, Kindler Syndrome and EBS with muscular dystrophy (1).

Optimal patient management requires a multidisciplinary approach, and revolves around the protection of susceptible tissues against trauma, use of sophisticated wound care dressings, aggressive nutritional support, and early medical or surgical interventions to correct whenever possible the extracutaneous complications. Prognosis varies considerably and is based on both EB sub-type and the overall health of the patient (1). It is already well known that patients affected by JEB have a susceptibility to urogenital involvement and if not treated they are prone to cancer development (2). However, we were unable to find any report of accompanying mesonephroid, intestinal and squamous metaplasia of the bladder in junctional EB in the English literature. This is the first report in a patient with junctional EB with these lesions. These patients should be followed regularly due to possible devastating complications. In these patients the risk of malignant transformation of bladder lesions should be kept in mind.
